# Myeloproliferative neoplasm with *ETV6-ABL1* fusion: a case report and literature review

**DOI:** 10.1186/1755-8166-6-39

**Published:** 2013-09-20

**Authors:** Katya Gancheva, Andres Virchis, Julie Howard-Reeves, Nick CP Cross, Diana Brazma, Colin Grace, Paul Kotzampaltiris, Fedra Partheniou, Elisabeth Nacheva

**Affiliations:** 1 Leukaemia Cytogenetics, Academic Haematology, UCL Medical School, Royal Free Campus, Rowland Hill Street, London NW3 2PF, UK; 2 Department of Haematology, Barnet and Chase Farm Hospitals NHS Trust, Barnet Hospital, Wellhouse Lane, Barnet, London, Hertforshire EN2 3DJ, UK; 3 Leukaemia Cytogenetics, Royal Free NHS Foundation Trust, Pond Street, London NW3 2QG, UK; 4 Faculty of Medicine, University of Southampton, Southampton, UK

**Keywords:** ETV6-ABL1, Atypical CML, Nilotinib, Resistance to imatinib

## Abstract

*ETV6-ABL1* is a rare gene fusion with oncogenic properties, reported so far in 28 patients presenting a variety of haematological malignancies associated with clinical outcome, including chronic myeloid leukaemia (CML), acute myeloid leukaemia (AML), acute lymphoblastic leukaemia (ALL) and chronic myeloproliferative neoplasm (cMPN). Here we report on a 46-year-old female who presented with Philadelphia negative CML, positive for the *ETV6-ABL1* fusion. Whole genome screening carried out with oligonucleotide arrays showed a subtle loss at 12p13 and cryptic imbalances within the 9q34.3 region in a highly unstable genome. FISH mapping with custom BAC probes identified two breakpoints 5 Mb apart within the 9q34 region, together with a break at 12p13. While FISH with commercial *BCR-ABL1* probes failed to detect any *ABL1* changes, the *ETV6* break-apart probe conclusively identified the *ETV6-ABL1* fusion thus determining the probe’s role as the primary diagnostic FISH test for this chimeric oncogene. In addition, we confirm the association of the *ETV6-ABL1* fusion with imatinib resistance reported so far in three other patients, while recording excellent response to the 2^nd^ generation tyrosine kinase inhibitor (TKI) nilotinib*.* In summary, we highlight the value of *ETV6* FISH as a diagnostic test and the therapy resistance of *ETV6-ABL1* positive disorders to imatinib.

## Introduction

CML is one of the most extensively studied human malignancies and was the first example of a disease, where the underlying molecular basis was a consistent chromosomal abnormality, the Philadelphia chromosome (Ph), that is the product of a balanced reciprocal translocation involving chromosomes 9 and 22. Over 95% of CML patients are found to carry the t(9;22)(q34;q11) and the resultant *BCR/ABL1* fusion gene as postulated by WHO classification (2008) [1]. Six other genes in addition to *BCR* can form a fusion product with *ABL1*. The chimeric proteins contain the kinase domain of ABL1 and are composed of the N-terminal part of the partner protein that includes a coiled-coil or a helix-loop-helix domain. Fusion genes with a break within intron 1 or 2 of *ABL1*, such as *BCR-ABL1*, *ZMIZ1-ABL1*, *EML1-ABL1* and *ETV6-ABL1*, carry transforming activity, while the *NUP214-ABL1* requires amplification to be efficient [2].

The *ETV6-ABL1* fusion was reported for the first time in a 22-month-old girl with ALL by Papadopoulos et al. in 1995 [3] and to date, this fusion gene has been reported in 28 cases of haematological malignancy [4-6]. Common characteristics of the *ETV6-ABL1* translocation appears to be eosinophilia, seen in 16 out of the 21 patients for which data was available, a 2:1 male predominance and seen in patients with ages varying between 8 months and 81 years. The *ETV6-ABL1* fusion is seen in a wide range of haematological malignancies: 5 patients had AML, 10 ALL and 3 with an MPN while the remaining 11 were described as having Ph negative CML, of which 3 presented in a blast crisis, summarised in Table 1 [3,4,7-24].

**Table 1:** Summary of published data for patients with etv6/abl1 fusion gene

**No**	**Dg**	**Sex**	**Age**	**TKI**	**Outcome**	**Eosinophilia**	**Transcript type**	**G banding**	**FISH/ETV6**	**FISH/ABL1**	**Genome features**	**Reference**
1	ALL	F	22 mo	No	Died		A	na	na	na	na	Papadopoulos et al, 1995 Cancer Research [3]
2	AML-M6	M	81	No	Died		B	t(9;12;14)(q34;p13;q22)/complex karyotype	na	na	na	Golub et al., 1996 Mol & Cellular Biology [7]
3	CML atypical	na	49	No	Died	Yes	B	na	na	na	na	Brunel et al., 1995 Leukemia [8]
4	CML	M	32	No	CR (>3Y)	Yes	B	46,XY,t(12;14)(p12;q11-13)	5’ETV6 on 9q34	**ABL1 3’/5’ on 9q34**	na	Andreasson et al., 1997 Genes, Chrom & Ca [9]
5	CML	M	59	No	Died	Yes	A,	46,XY,del(6)(p21),?t(9;12)(q34;p12)	5’ETV6 on 9q34	**ABL1 3’/5’ on 9q34**	na	Van Limbergen et al., 2001 Genes, Chrom & Ca [10]
6	T/ALL	M	4	No	Died	Yes	A,B	47,XXYc,del(6)(q15q23)	5’ETV6 on 9q34	**ABL1 3’/5’ on 9q34**	na	Van Limbergen et al., 2001 Genes, Chrom & Ca [10]
7	AML-M6/ CML/MBC	M	38	Yes	Died	na	A,B	46,XY	na	3’ABL1 on 12p	karyotype evolution	O’Brien et al., 2002 Blood [11]
8	CML	M	53	No	CR (>6Y)	Yes	A,B	46,XY	na	**ABL1 3’/5’ on 9q34**	na	Lin et al., 2002 Leukemia [12]
9	CML	F	44	Yes	CR (>6 M)	Yes		46,XX,t(9;12)(q34;p13)	5’-3’ETV6 on 9q34	**ABL1 3’/5’ on 9q34**	na	Keung et al., 2002 Ca Gen &Cytogen [13]
10	AML-M1	M	29	No	CR (>20 M post SCT)	Yes	A	46,XY,t(8;12)(p21;p13)	5’ETV6 on 8p21	3’ABL1 on 8p21	na	La Starza et al., 2002 Haematologica [14]
11	AML-M1 post RAEB	M	48	No	Died	Yes	B	46,XY,t(9;12)(q34;p13)	na	3’ABL1 on 12p	karyotype evolution	La Starza et al., 2002 Haematologica [14]
12	CML-MBC	M	36	Yes	Died	Yes	B	45,XY-7,t(9;12)(q34;q13)	na	3’ABL1 on 12p	karyotype evolution	Barbouti et al., 2003 Br J Haematol [15]
13	CML-LBC	M	72	Yes	CR (>12 M)	na	B	46,XY,t(12;17)(p11.2;p11.2)	5’ETV6 on 17p	3’ABL1 on 17p		Tirado et al., 2005 Ca Gen & Cytogen [16]
14	cMPN	F	65	No	Died	Yes	A,B	46,XX ,t(5;9)(q13;q34)	na	3’ABL1 on 12p	karyotype evolution	Meyer-Monard et al., 2005 Leukemia [17]
15	cMPN	M	57	No	CR (>15Y)	Yes	na	46,XY	na	3’ABL1 on 12p	na	Mozziconacci et al., 2007 Amer J of Haematol [18]
16	ALL	M	30		Died		A,B	45,XY,del(1)(q42),-9,-13,add(16)(p1?3),+mar	na	3’ABL1 on 12p	p16 loss	Baeumler et al., 2008 Ca Gen & Cytogen [19]
17	CML	F	24	Yes	CR (>7 M)	Yes	A	46,XX	5’ETV6 on 9q34	**ABL1 3’/5’ on 9q34**	loss ASS-ABL1 exon1	Kawamata et al., 2008 Genes,Chrom & Ca [20]
18	cMPN	F	61	Yes	CR (>3Y)	Yes	na	46,XX	5’-3’ETV6 on 12p	3’ABL1 on 12p	na	Nand et al., 2009 Leuk Research [21]
19	CML atypical	M	79	Yes	Died	Yes	na	46,XY	5’-3’ETV6 on 12p	3’ABL1 on 12p	na	Kelly et al., 2009 Ca Gen &Cytogen [22]
20	ALL	F	33		Died	No	A,B	46,XY, der(1)t(1;?)	na	normal	p16/p15 loss	Zuna et al., 2010 Genes,Chrom & Ca [4]
21	ALL	M	5	No	CR (>24 M)	Yes	A,B	46,XY	na	3’ABL1 on 12p	p16/p15 loss	Zuna et al., 2010 Genes,Chrom &Ca [4]
22	ALL	M	8 mo	Yes	Died	No	A,B	46,XX,t(8;9;12)(p12;q34;p13)	na	3’ABL1 on 12p	na	Zuna et al., 2010 Genes,Chrom & Ca [4]
23	ALL	F	8	Yes	CR( >10 M)	na	A,B	46.XX	na	3’ABL1 on 12p?	na	Malone A et al., 2010 Br J Haematol [6]
24	CML	M	36	Yes	CR(>5Y)	No		46,XY, t(9;12)(q34;p13)	na	3’ABL1 on 12p	normal UTX, ASXL1, EZH2, TET2 & IDH1/2	Perna et al., 2011 Haematologica [24]
25	T/ALL	na	na	na	na	na	na	na	na	na	na	Zhou et al., 2012 Annual Haematology [5]
26	T/ALL	na	na	na	na	na	na	na	na	na	na	Zhou et al., 2012 Annual Haematology [5]
27	AML	M	52	No	Died	Yes	B	46,XY[20]	5’-3’ETV6 on 12p	3’ABL1 on 12p	normal FLT3, cKit & NPM1	Park J et al., 2013 Acta Haematologica [23]
28	B/ALL	F	25	No	Died	No	A,B	46,XX,del(9)(p22), der(10)t(9;10)(q22;p15)	5’-3’ETV6 on 12p	3’ABL1 on 12p	na	Park J et al., 2013 Acta Haematologica [23]
29	CML	F	52	Yes	CR > 12 M	Yes	A,B	46,XX,t(9;12)(q34;p13)	5’ETV6 on 9q34	**ABL1 3’/5’ on 9q34**	37 CNAs	Current study

*Abbreviations*: *ALL* Acute lymphoblastic leukemia, *AML* Acute myeloid leukemia, *CML* Chronic myeloid leukemia, *MPN* Myeloproliferative disease, *MBC* Myeloid blast crisis, *LBC* Lymphoid blast crisis, *F* Female, *M* male, *CR* Cytogenetic remission, *Na* not available, *CNAs* Copy number aberrations.

The structure of the ETV6-ABL1 oncoprotein is similar to that of BCR-ABL1, as evidenced by the fact that both fusion products lead to activation of the non-receptor tyrosine kinase ABL1 with initiation of similar downstream pathways effecting cellular survival, growth rate and independence as well as transforming capacity [25].

The existence of two different transcripts, type A and B, is evidence for alternative splicing. Type A transcript includes the first four exons of *ETV6*, fused to exon 2 of *ABL1*, while type B includes exons 1 to 5 of *ETV6* fused to *ABL1* exon 2. The difference between the two transcripts at protein level is the presence or absence of a direct binding site for the SH2 domain of the GRB2. Million et al [26] showed that the GRB2 binding site in the ETV6-ABL1 product has several functions - from activation of the GAB2, PI3-kinase, and ERK-MAPK pathways to transformation of fibroblasts and B-lymphoid cells all of which are required for efficient induction of CML-like MPN. Furthermore, they demonstrated that the absence of *ETV6* exon 5 leads to a slightly lower tyrosine kinase activity of the type A ETV6-ABL1 protein, although both kinases are as catalytically active as is the BCR-ABL1 product.

Since the *ETV6* and *ABL1* genes have an opposite orientation to the chromosome centromere, the formation of a fusion requires at least three chromosomal breaks to be generated [2]. This could be the reason for the well-documented low incidence of *ETV6-ABL1* fusion cases. Furthermore little is known regarding the effect of treatment with tyrosine kinase inhibitors on *ETV6-ABL1* fusion positive haematological conditions.

Here we report a female patient, who presented with Philadelphia negative CML and t(9;12)(q34;p13) as a sole bone marrow karyotype abnormality leading to *ETV6-ABL1* fusion formation. Located on der(9)t(9;12), this fusion oncogene is shown to result from multiple events within a 5.6 Mb region at 9q34.12-q34.3, which escapes detection by commercial *BCR-ABL1* FISH probes. Importantly, full cytogenetic and molecular remission was achieved only after second generation TKI treatment thus associating the *ETV6-ABL1* fusion with TKI resistance.

## Case report

In August 2011 a 46-year-old female presented with fatigue, a leucocytosis and thrombocytosis. Blood film examination was suggestive for CML with a neutrophilia, myelocyte peak, basophilia and also of note a eosinophilia of 2.5 × 10^9^/l. The aspirate morphology reported myeloid hyperplasia with reduced erythropoiesis, and increased eosinophilic and basophilic precursors. Histopathology reported a myeloproliferative neoplasm suggestive for CML chronic phase, with a markedly hypercellular bone marrow and myeloid hyperplasia with a left shift and loss of normal architecture. Erythroid activity was markedly reduced - only scattered CD71 positive cells were present. CD34 and CD117 show less than 5% blasts. G-banding identified a balanced t(9;12)(q34;p13) in 10/10 metaphases and interphase FISH was positive in 92% of cells analysed. RT-PCR revealed the presence of *ETV6-ABL1* fusion mRNA, while BCR-ABL1 was not detected. Such cases are not adequately covered by the WHO classification of myeloproliferative disorders and could be categorized as a ? CML variant.

Imatinib therapy achieved a complete haematological response (CHR) after 4 weeks and at 3 months, interphase FISH on the peripheral blood was positive in 37/200 cell (18.5%). At 5 months thrombocytosis had recurred with the reappearance of a mild neutrophilia, basophilia and eosinophilia at 6 months. FISH analysis was positive in both interphase (50/200) and metaphase (6/20) cells and residual disease was confirmed by nested RT-PCR. She was switched to nilotinib, once again achieving a CHR after 4 weeks, and a complete cytogenetic response (CCyR) at 3 months. Major molecular response (MMR) was also achieved and in the subsequent specimens taken at 10, 12 and 18 months, *ETV6-ABL1* mRNA was no longer detectable with nested RT-PCR. Both CCyR and MMR are sustained to date 22 months from diagnosis.

## Methods

### (i) Cell Culture and chromosome studies

Bone marrow (BM) and peripheral blood (PB) cell samples were cultured in RPMI medium following routine protocols as previously described [11]. High-resolution chromosome banding analysis was carried out and ISCN 2013 nomenclature was used to describe chromosome abnormalities [18].

### (ii) FISH and molecular cytogenetic analysis

Fluorescence in situ hybridization (FISH) investigations were performed using commercially available *BCR-ABL1* probes (dual fusion probe, *Vysis, USA*) and *ETV6* (break-apart probe *Vysis, USA)*. Chromosome mapping of the 9q34 regions was carried out with a range of BAC clones *(BACPAC Resources, USA).* For disease monitoring a double fusion dual colour FISH probe was made with BAC clones covering the regions of *ABL1* (RP11-57C19 and RP11-83 J21), *ETV6* (RP11-356 K6, RP11-418C2 and RP11-639O1) and *NOTCH1* (RP11-707O3 and RP11-678D10) genes.

### (iii) RT-PCR

Total RNA was extracted from the cell culture using Trizol reagent (*Life Technologies, USA).* Purified RNA was reverse transcribed, quality controlled and tested for *BCR-ABL1* as described previously [27]*ETV6-ABL1* was amplified in pretreatment samples by single step PCR using primers Tel3F2 (5’-CGCTATCGATCTCCTCATTCA-3’) and CA3-b: (5’-ACACCATTCCCCATTGTGAT-3’). Two products were amplified, corresponding to a fusion *ETV6* exon 4 to *ABL1* exon 2 and *ETV6* exon 5 to *ABL1* exon 2. Remission samples were tested by nested PCR using primers TEL3F: (5’-CTGCTGACCAAAGAGGACTT-3’) and CA3-: (5’-TGTTGACTGGCGTGATGTAGTTGCTTGG-3’) in the first step and Tel32F2 + CA3-b in the second step. Dilution of pretreatment cDNA into negative control DNA indicated a sensitivity of detection of 10^-4^.

### (iv) Array Comparative Genomic Hybridisation (aCGH)

Array CGH analysis was performed with a high-density customised 400 K oligonucleotide platform enriched with probes covering the coding regions of oncogenes (ID 027193, *Agilent technologies, USA*) following the manufacturer’s protocol. In brief, 1500 ng genomic test DNA was hybridised against a reference of a pooled DNA samples collected from PBMC of 6 - 8 disease free individuals (Promega, UK). We used Genomic Workbench v.6 (Agilent technologies, USA) for data analysis as previously [28].

## Results

### (i) Philadelphia chromosome and BCR/ABL1 fusion are absent

**Figure 1:**
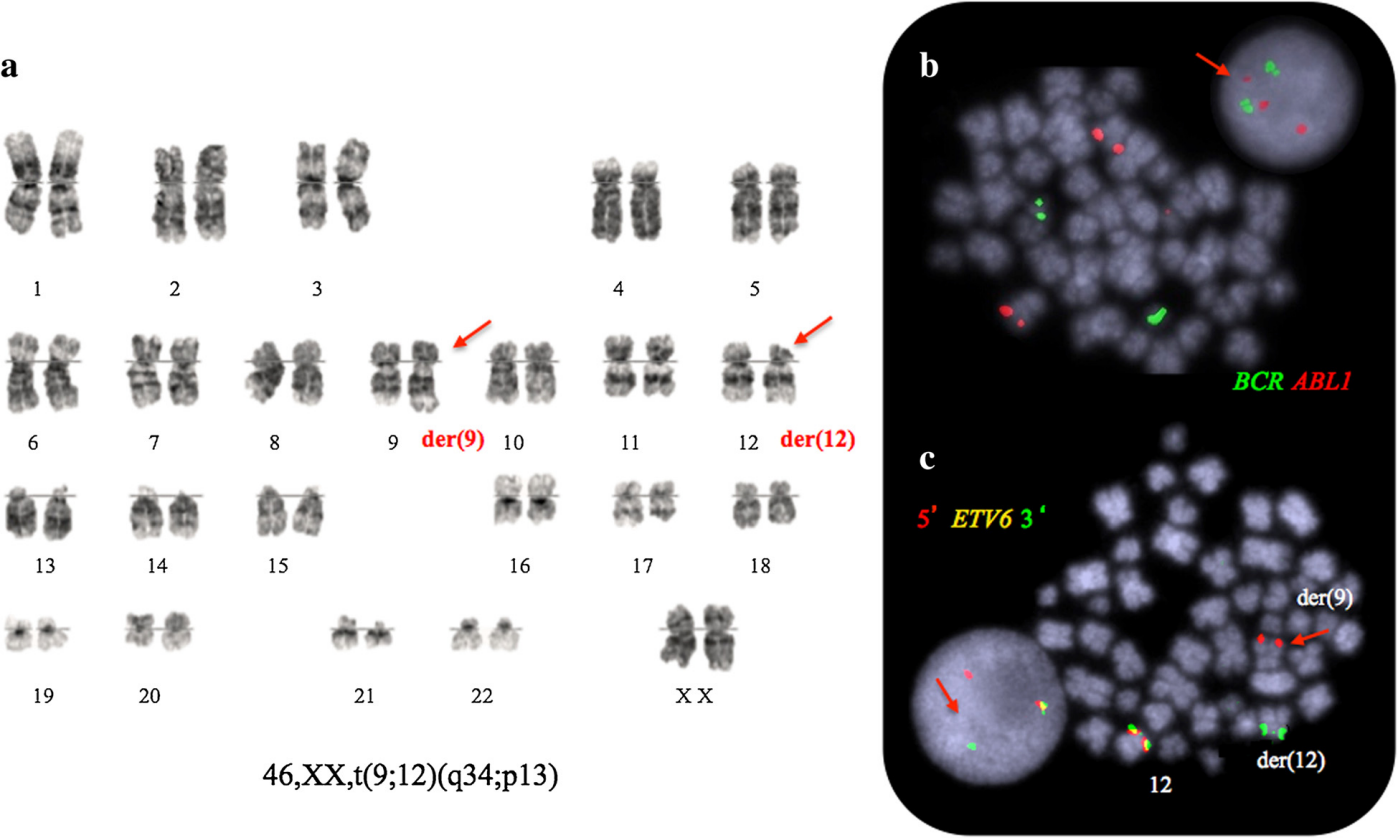
**Representative Philadelphia negative metaphase bone marrow cell with a t(9;12)(q34;p13) translocation. (a)** G-banded karyotype; **(b)** FISH analysis demonstrates lack of *BCR-ABL1* fusion but reveals a small third signal from the ABL1 probe (arrow) and; **(c)** The ETV6 split signal (arrowed in red) on der(9)t(9;12) from the break-apart FISH probe shows the gene rearrangement and confirms G banding results. The red/green fusion signal marks the normal gene.

G banding analysis of bone marrow cultures found both homologues of chromosome 22 intact, but identified a translocation of the long arm of chromosome 9 due to t(9;12)(q34;p13) (Figure 1a). FISH analysis using a dual fusion, dual colour *BCR-ABL1* probe (Vysis) confirmed the absence of a fusion gene and hence the Philadelphia chromosome. However, some 21% of the interphase BM cells were found to harbour a third, albeit significantly smaller, *ABL1* signal found to be absent from the metaphases. The *ABL1* part of the D-FISH probe is 650 Kb long and any break upstream of exon 2 would result in two different in size segments. The proximal fragment covering the *ASS* – *ABL1* exon 2 region is larger (~500 Kb) and will remain on der(9)t(9;12). However, the FISH signal from the smaller distal segment (~150 Kb) encompassing *ABL1* exon 2 to 11 was not found on the der(12)t(9;12) chromosome as expected, although detected in some of the interphase cells (Figure 1b).

### (ii) Formation of the ETV6-ABL fusion at der (9)t(9;12)(q34;p13)

**Figure 2:**
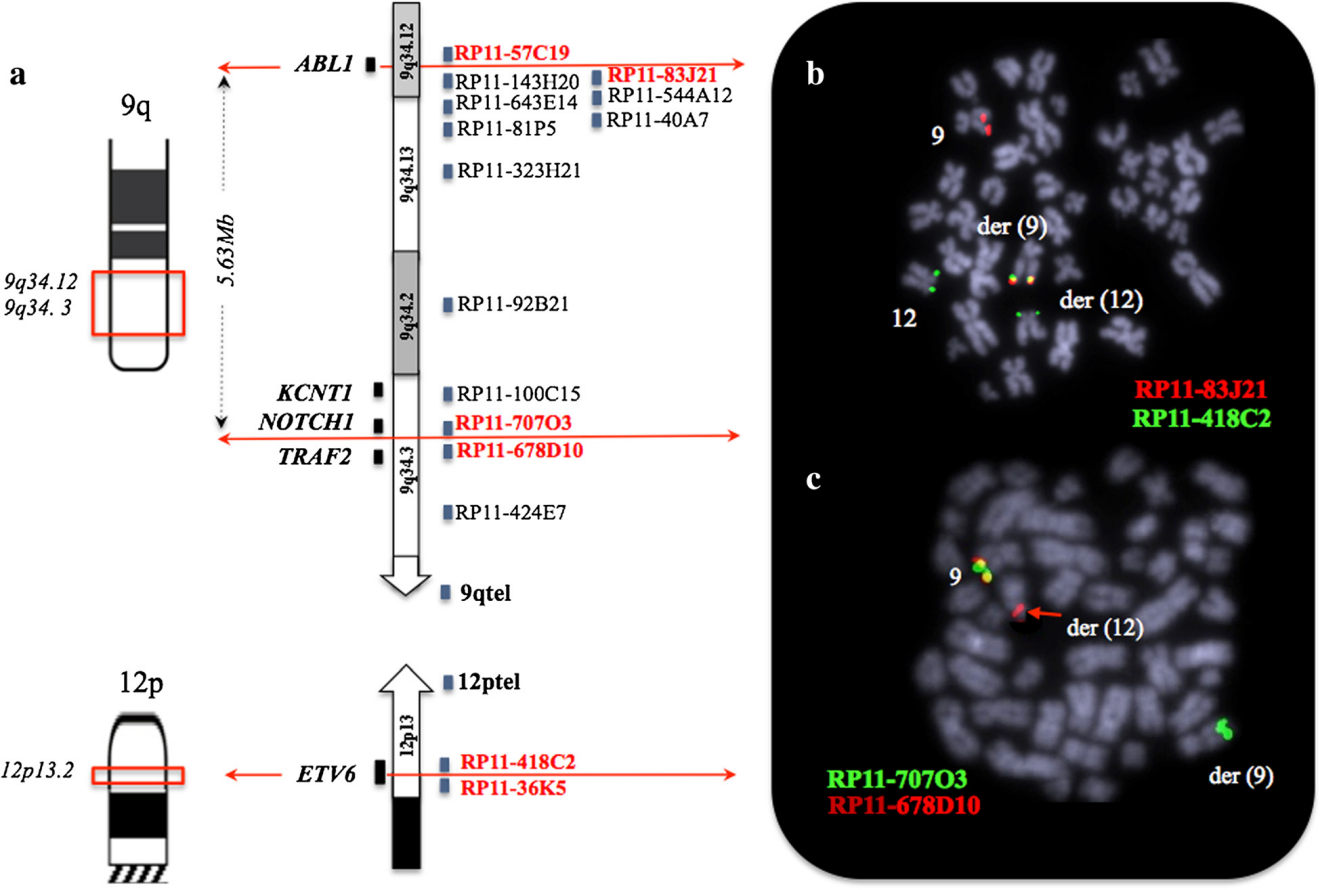
**FISH mapping of 9q34 and 12p13 regions in t (9;12)(q34;p13). (a)** Diagram of 9q34 (top) and 12p13 chromosome regions with a map of the BAC clones used for FISH analysis. Red arrows indicate breakpoint positions in 9q34.12, 9q34.3 and 12p13.2; **(b)** Exons 2-11 of the *ABL1* (RP11-83 J21 in red) on der(9)t(9;12) co-localizing with the 5’ part of the *ETV6* gene (RP11-418C2 in green) and **(c)** The break at 9q34.3, 5.63 Mb distal to *ABL1*, is flanked proximally by RP11-707O3 (green) which houses the *NOTCH1* gene and remains in der(9)t(9;12) and distally by RP11-678D10 (red) which has moved to der(12)t(9;12) (arrowed).

**Figure 3:**
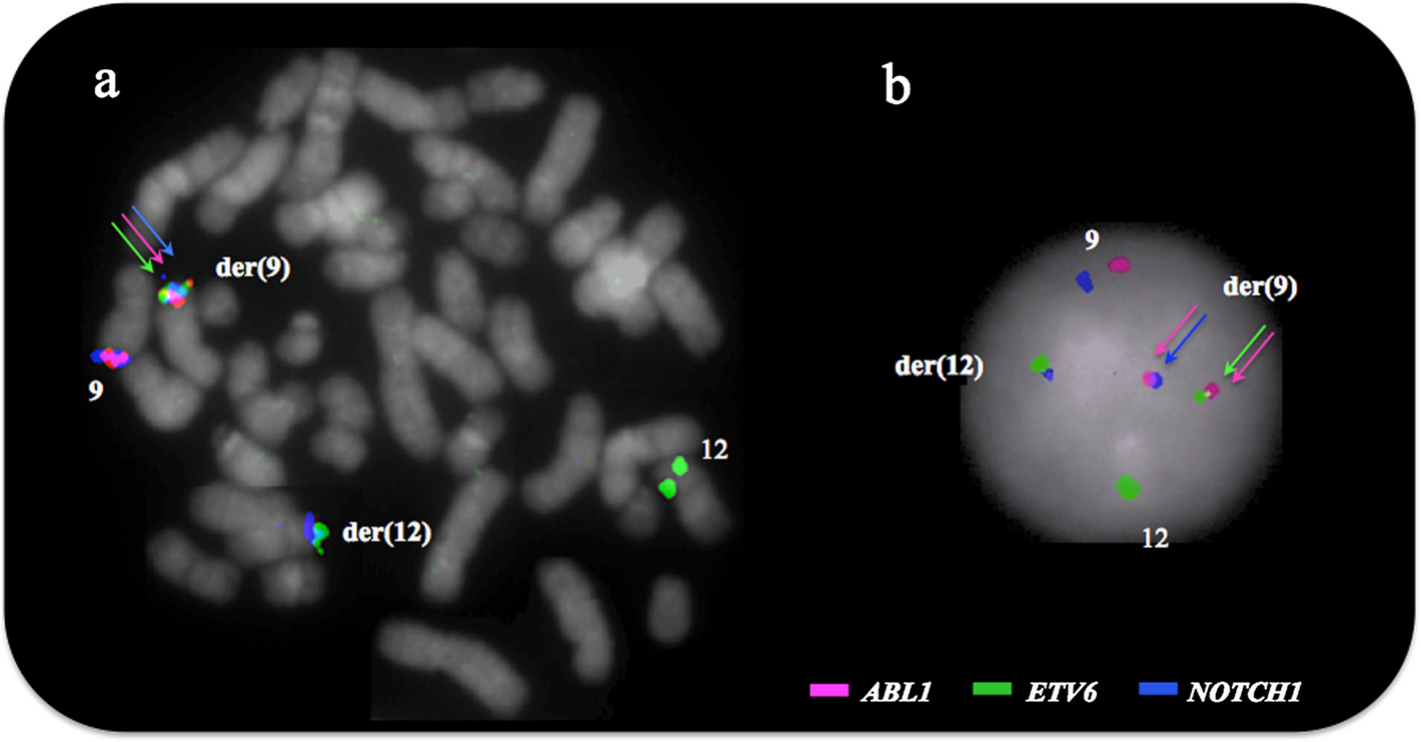
**Representative cells with FISH signals showing the cryptic three way rearrangement of *ABL1***, ***NOTCH1 *and *ETV6 *associated with t(9;12)(q34;p13).** BAC clones covering the regions of interest are as follows: *ABL1* (RP11-83 J21 & RP11-57C19, in red), *NOTCH1* (RP11-707O3&RP11-678D10, in blue) and *ETV6* (RP11-418C2 & RP11-36 K5, in green) **(a)** FISH signals from all three genes - *ETV6*, *ABL1* and *NOTCH1* - cluster at der(9)t(9;12) in a metaphase cell (arrows) **(b)** in a non-dividing cell the *ETV6* (exons 1-5)/*ABL1* (exons 2-11) fusion(green/red arrow) is separated from the co-localized *ABL1* (exons 1b-2) and *NOTCH1* signals (red/blue arrows); while *ABL1* and *NOTCH1* signals (~5.6 Mb apart) mark the normal 9 homologue and signals from *ETV6* (green) and RP11-678D10 (downstream of *NOTCH1*, blue) co-hybridize at der(12)t(9;12).

A commercial *ETV6* break-apart probe (*Vysis*) yielded a split signal located on der(9)t(9;12) in all dividing and 92% of the interphase BM cells demonstrating the rearrangement (Figure 1c). RT-PCR using published primers identified both type A and B transcripts of the *ETV6-ABL1* fusion thus confirming ABL1 involvement [10]. However, such transcripts are unlikely to be a direct result of the t(9;12)(q34;p13) because of the different orientation of the *ETV6* and *ABL1* genes. Furthermore, extensive research has already shown [4,10] that the formation of a functional *ETV6-ABL1* fusion gene requires additional genome aberrations. Our failure to detect *ABL1* signals on the der(12)t(9;12) in metaphase cells prompted us to search the 9q34 region by FISH 'walking’ for multiple events that could facilitate the formation of the *ETV6-ABL1* fusion (Figure 2). We detected a single break within 12p13 region and translocation of the 5’ part of the *ETV6* gene (exon 1-5) some 40 Kb distal of the *NOTCH1* gene (Figure 1c). This was shown by the presence of the BAC RP11-707O3 on der(9)t(9;12), while the distally located RP11-678D10 is moved to der(12)t(9;12). This FISH signal pattern suggests that *ETV6* exons 1 - 5 sequences are located distally to *ABL1* (Figure 2*,* Additional files 1 &2). Indeed the *ABL1* gene appears unaffected since FISH analysis of dividing cells shows both BAC clones that encompass the entire gene (i.e. RP11-57C19 and RP11-83 J21) within the 9q34 region of der(9)t(9;12). In contrast, FISH using either custom 9q34 BAC clones or commercial *BCR-ABL1* probes on interphase cells where the chromatin is less condensed allowing the discrimination of closely spaced DNA probes, revealed a third *ABL1* signal indicating breakage. The application of a cocktail of BAC clones in a three colour FISH experiment covering all relevant breakpoints in 12p13, 9q34.12 and 9q34.3, showed that in interphase cells the *ETV6-ABL1* fusion is a considerable distance away from the proximal part of *ABL1* (exon 1b-2) and the neighbouring *NOTCH1* gene (Figure 3*, arrows*), while in metaphase cells their discrimination is impossible. The *ETV6-ABL1* fusion (both type A and type B transcripts) was confirmed by the RT-PCR and shown to occur after the break in the exon 1a-2 region of *ABL1* and exon 5 of *ETV6* as expected. Whole genome scanning using oligonucleotide arrays revealed a total of 37 cryptic copy number aberrations (CNAs), mostly (24/37) gains *(*Additional file 3*)*. A cluster of CNAs was found within 9q33-qter including extra copy of the *DAB2IP* (a tumour suppressor gene with a recognised role in breast and prostate cancer) and within the regions flanking *NOTCH1*. A cryptic 140 bp loss at chr12: 12,045,340 – 12,045,484 was found at the breakpoint in the *ETV6* gene (arrowed in Additional file 3). Other affected genome sites include the *TNK2* gene at 3q29, ~3 Mb gain at 8q24.3 that involves *TOP1MT* and *MAPK15* genes among others and extra copies of the *MLL* gene at 11q23 as well as *MLLT1* at 19p13. Among the loci found deleted are transcription co-activator gene *CREBBP* at 16p13.3 and *CBFb* at 16q22 (Additional file 3). It is noteworthy that only 2 of the 37 CNAs in this molecular karyotype are reported polymorphic imbalances, the rest represent cryptic secondary changes indicative of genome instability.

## Discussion

*ETV6* is one of the six genes known to form fusion chimeric transcripts with *ABL1*. As a rule, the fusion gene results from joining the 3’sequences of *ABL1* with the 5’ end of the partner genes. While most of these fusion genes are associated with a specific type of leukaemia, *BCR-ABL1* and *ETV6-ABL1* are found in a wide spectrum of clinically and morphologically different malignant blood conditions [2]. In spite of their heterogeneity these conditions are likely to share a common progenitor cell, the pluripotent stem cell, since both chimeric genes activate similar transduction pathways with similar transforming activity [29].

The *ETV6-ABL1* fusion gene is a truly rare event. So far there are 29 cases including the present study published after the first report in 1995 by Papadopoulos et al. (summarised in Table 1). The rarity of this fusion is due to the opposite transcriptional orientation of the two genes relative to the centromeres, which would require at least two events to form an in-frame fusion transcript. It is therefore not a surprise that the chromosome rearrangement producing the fusion gene often remains hidden. Perhaps the *ETV6-ABL1* fusion is not as rare as the literature suggests since there are no satisfactory commercial FISH probes available for the detection of the *ETV6-ABL1* fusion. In dividing cells with t(9;12)(q34;p13), the dual colour/dual probe *BCR/ABL1* set (D-FISH) should indicate an *ABL1* rearrangement by producing a third *ABL* signal on der(12) while *BCR* remains intact (Figure 1b). Unfortunately this abnormal signal pattern was not seen in metaphase cells in nearly a third of cases (27%) where both parts of the rearranged *ABL1* remain within the 9q34/qter (Table 1). The situation is worse in interphase cells where the third signal may remain unaccounted for due to its small size as illustrated in our case and seen in a further 6 reports with *ETV6-ABL1* fusion (Table 1). Analysts using widely available BCR-ABL1 commercial assays on interphase cells may regard the disproportionately small third signal from the *ABL1* probe as 'noise’. In contrast, FISH with the *ETV6* break-apart (BA) probe will produce a split (third) signal at the der(9) chromosome that is easy to detect in interphase cells of fusion carriers (see Figure 2c). Therefore FISH screening with *ETV6* (BA) probe of samples suspected for CML but negative for *BCR-ABL1* rearrangement by FISH, provides a reliable way to reassess the 'rarity’ of this fusion gene. Indeed two recent studies [4,5] have indicated that the incidence of *ETV6-ABL1* fusion transcript may well be around 1%, thus confirming our concerns that the occurrence of this translocation in haematological diseases is underestimated.

**Figure 4:**
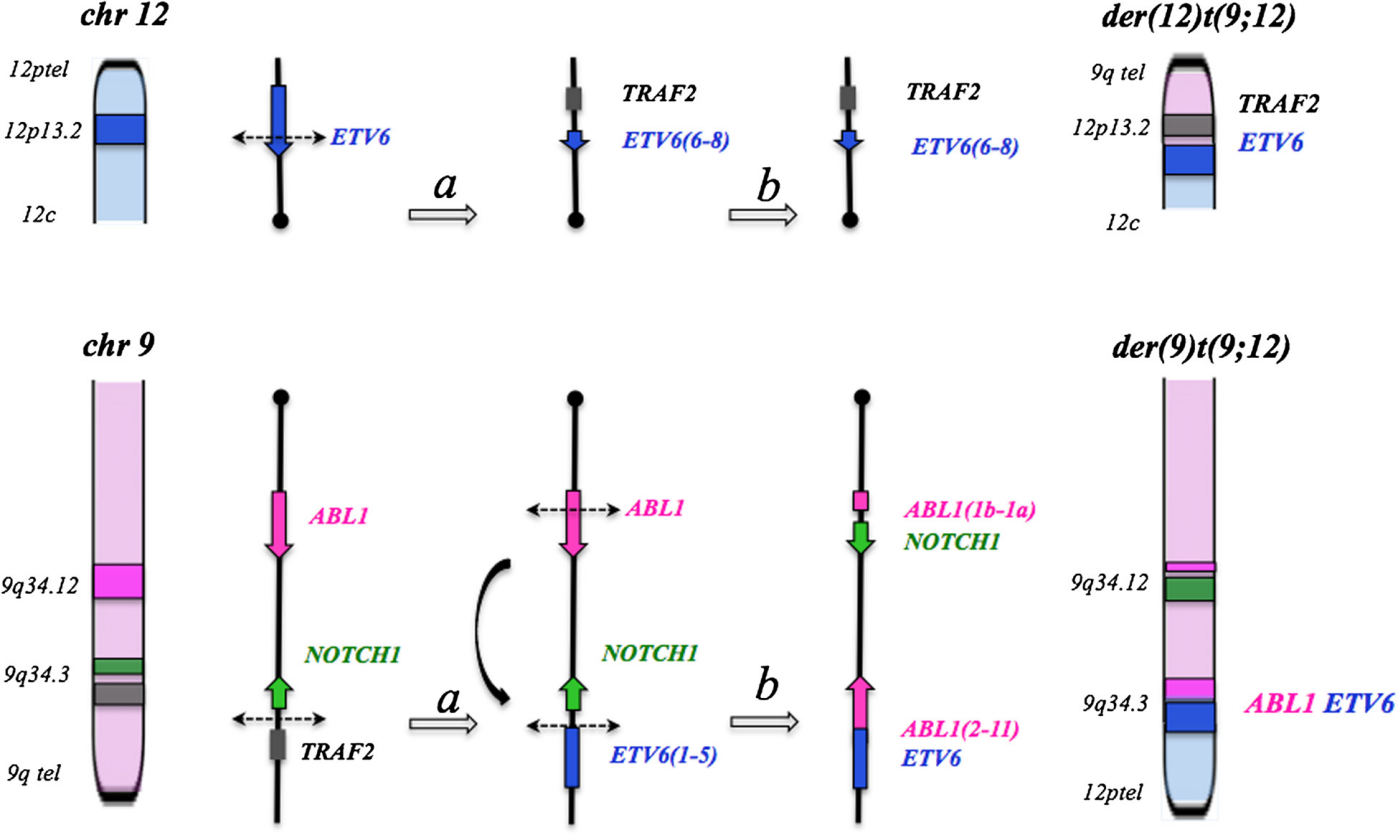
**Formation of a fusion between the *ABL1 *and *ETV6 *genes with opposite chromosomal orientation via two events: (i) firstly, a balanced t(9;12)(q34;p13) results in the juxtaposition of part of the *ETV6 *gene (exons 1-5) in the vicinity of *NOTCH1 *at 9q34.3 on der(9)t(9;12) while *ABL1 *remains intact followed by (ii) an inversion within the 9q34 segment of der(9)t(9;12) after breaks at 9q34.12 (within *ABL1*, upstream of exon 2) and 9q34.3.** This leads to formation of an *ETV6-ABL1* fusion gene but leaves the *NOTCH1* gene intact.

There are few descriptions in the literature of in-frame fusion products involving genes with different orientations. As a rule, formation of these fusion genes is associated with more than one event and more than the two breaks that are necessary for a classical reciprocal translocation. For example, Van Limbergen and colleagues [10] suggested two alternative mechanism. Our findings support a complex model (see Figure 4*)* that incorporates some of the suggested changes and highlight the fact that the *ETV6-ABL1* fusion resides on der(9)t(9;12) in nearly a third of the cases thus rendering FISH with *ABL1* probes unfit for purpose.

Million et al. [30] demonstrated a striking similarity between the *ETV6-ABL1* and *BCR-ABL1* induced leukaemia in mouse models, the only difference being the latency period. They found that the ETV6-ABL1 fusion protein is significantly more active compared to the p210 BCR-ABL1. Importantly, both fusion proteins were shown to have similar helix-loop-helix domains, which is fused to the kinase domain of *ABL1* and implicated in protein oligomerisation process [31]. Indeed *ETV6-ABL1* positive patients respond to treatment with tyrosine kinase inhibitors (TKI) as expected. Kawamata et al., reported a favourable response to imatinib in chronic phase CML with normal karyotype and *ETV6-ABL1*[20]. However, the inhibitory effect of imatinib was short lived and unable to induce a complete remission in two further CML cases [13,15]. Similarly, Nand et al., [21] reported an *ETV6-ABL1* fusion positive patient with a myeloproliferative disorder who developed morphologic and cytogenetic relapse after 17 months on imatinib but achieved complete remission on a second second-generation TKI (nilotinib). After an initial haematological response to imatinib 400 mg daily our patient, who presented with typical chronic phase CML and t(9;12)(q34;p13) as sole karyotype abnormality, progressed at 3 months; whereas nilotinib achieved a prompt CCyR, followed by an MMR 16 months after diagnosis, which is sustained to date. The mechanism of the imatinib resistance reported here and by others is still unexplained. Although the long-term response to second generation TKIs remains to be determined, their implementation as first line therapy in *ETV6-ABL1* (+) disorders is well supported.

## Conclusion

In summary, we highlight two main features of *ETV6-ABL1* positive disorders: firstly, the diagnostic value of FISH with the ETV6 (BA) probe and secondly, while this chimeric oncogene is associated with poor long term response to imatinib, our patient achieved a sustained response to nilotinib.

## Consent

Written informed consent was obtained from the patient for publication of this Case Report and any accompanying images. A copy of the written consent is available for review by the Editor-in-Chief of this journal.

## Competing interests

The authors declare that they have no competing interests.

## Authors’ contributions

KG carried out the molecular genetic studies, participated in the design of the study and drafted the manuscript. AV provided the clinical care and treatment and edited the manuscript. NC carried out the diagnostic RT-PCR analysis and edited the manuscript; JHR, DB, PK and FP carried out the diagnostic G-band and FISH analysis together with response to treatment. CG coordinated the array analysis and edited the manuscript. EN conceived, designed and coordinated the study. All authors read and approved the final manuscript.

## Supplementary Material

Additional file 1: Table S1 Addresses of the BAC clones. Click here for file

Additional file 2: Table S2 Gene addresses. Click here for file

Additional file 3: Figure S1 Array CGH results. Click here for file
