# Supplementary material for: Myeloproliferative neoplasm with ETV6-ABL1 fusion: a case report and literature review
Source: Mol Cytogenet. 2013 Sep 20;6:39. doi: 10.1186/1755-8166-6-39 (PMC3853649; doi:10.1186/1755-8166-6-39)
Supplement: Additional file 1: Table S1 — Addresses of the BAC clones. [file 1755-8166-6-39-S1.pdf]

Table S1: Addresses of the BAC clones used

| BAC clone name | Bands | Address | Length |
|---|---|---|---|
| **RP11-57C19** | 9q34.11 - 9q34.12 | Chr9: 133480545 - 133654007 | 173,462 bp |
| **RP11-83J21** | 9q34.12 | Chr9: 133,652,008-133,828,473 | 176,465 bp |
| **RP11-143H20** | 9q34.12 - 9q34.13 | Chr9: 133,851,940-134,028,561 | 176,622 bp |
| **RP11-544A12** | 9q34.12 - 9q34.13 | Chr9: 133,965,251-134,162,272 | 197,021 bp |
| **RP11-643E14** | 9q34.13 | Chr9: 134,156,635-134,334,397 | 177,763 bp |
| **RP11-40A7** | 9q34.13 | Chr9: 134,384,996-134,560,769 | 175,773 bp |
| **RP11-323H21** | 9q34.13 | Chr9: 134,558,770-134,738,990 | 180,220 bp |
| **RP11-81P5** | 9q34.13 | Chr9: 135,085,896-135,278,660 | 192,764 bp |
| **RP11-92B21** | 9q34.2 - 9q34.3 | Chr9: 137,291,566-137,467,914 | 176,319 bp |
| **RP11-100C15** | 9q34.3 | Chr9: 138,540,281-138,727,423 | 187,143 bp |
| **RP11-413M3 (RP11-611D20)** | 9q34.3 | Chr9: 139,437,333-139,438,287 | 955 bp |
| **RP11-678D10** | 9q34.3 | Chr9: 139,616,733-139,799,803 | 183,071 bp |
| **RP11-424E7** | 9q34.3 | Chr9: 140,885,563-141,087,415 | 201,853 bp |
| ***RP11-707O3*** | 9q34.3 | Chr9: 139,250,847-139,453,805 | 202,959 bp |
| ***RP11-36K5*** | 12p13.2 | Chr12: 12,088,525-12,274,586 | 186,062 bp |
| ***RP11-418C2*** | 12p13.3 | Chr12: 11,916,329-12,086,277 | 169,949 bp |
