# Supplementary material for: Myeloproliferative neoplasm with ETV6-ABL1 fusion: a case report and literature review
Source: Mol Cytogenet. 2013 Sep 20;6:39. doi: 10.1186/1755-8166-6-39 (PMC3853649; doi:10.1186/1755-8166-6-39)
Supplement: Additional file 2: Table S2 — Gene addresses. [file 1755-8166-6-39-S2.pdf]

Table S2: Genes addresses

Gene	Address	Lenght
**ABL1**	Chr9: 133,710,831-133,763,062	52,232 bp
**ETV6**	Chr12: 12,022,358-12,268,261	245,904 bp
**NOTCH1**	Chr9: 139,396,889-139,408,003	11,115 bp
**TRAF2**	Chr9: 139,793,193-139,820,353	27,761 bp
