# Supplementary figures and images for: Myeloproliferative neoplasm with ETV6-ABL1 fusion: a case report and literature review
Source: Mol Cytogenet. 2013 Sep 20;6:39. doi: 10.1186/1755-8166-6-39 (PMC3853649; doi:10.1186/1755-8166-6-39)

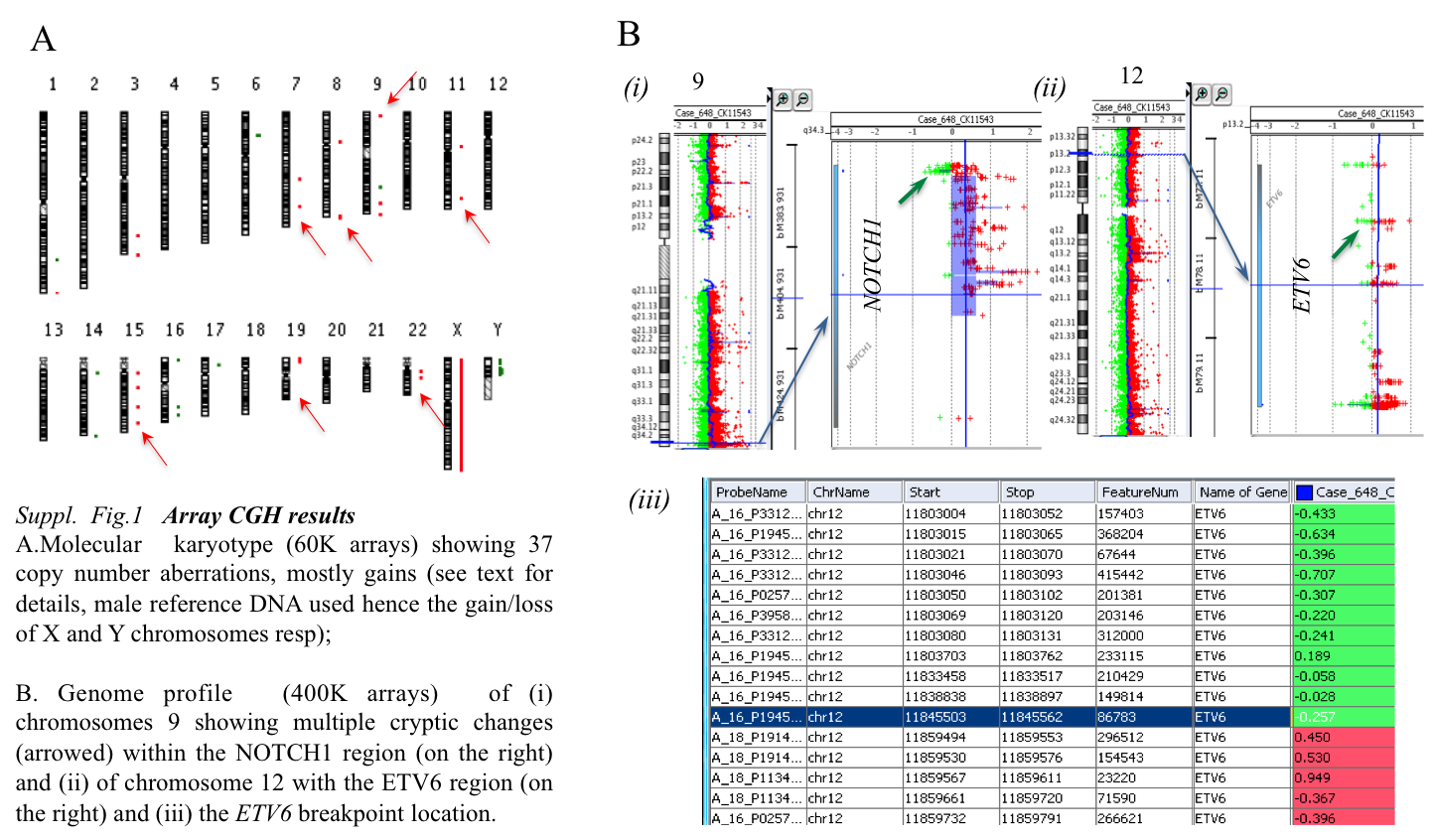

Supplement: Additional file 3: Figure S1 — Array CGH results. [file 1755-8166-6-39-S3.png]
